# Clonal hematopoiesis of indeterminate potential and cancer immunotherapy: a host-intrinsic immune-inflammatory modifier of treatment efficacy and toxicity

**DOI:** 10.3389/fonc.2026.1966718

**Published:** 2026-09-23

**Authors:** Longjun Huang, Jiahao Sheng, Luyang Chi, Yehan Xu, Yun Wang

**Affiliations:** 1 Department of Oncology and Hematology, Dongzhimen Hospital, Beijing University of Chinese Medicine, Beijing, China; 2 Beijing University of Chinese Medicine, Beijing, China; 3 The First Clinical Medical College of Zhejiang Chinese Medical University, Hangzhou, Zhejiang, China; 4 Department of Oncology, The First Affiliated Hospital of Zhejiang Chinese Medical University (Zhejiang Provincial Hospital of Chinese Medicine), Hangzhou, Zhejiang, China

**Keywords:** biomarkers, causality, chimeric antigen receptor T cells, clonal hematopoiesis of indeterminate potential, immune checkpoint inhibitors, immune-related adverse events, inflammation, tumor microenvironment

## Abstract

Clonal hematopoiesis of indeterminate potential (CHIP), the age-related expansion of hematopoietic stem cells carrying leukemia-associated somatic mutations, is common in patients with cancer and is increasingly detected during tumor-only genomic profiling and cell-free DNA sequencing. In this narrative review, we assess epidemiological, mechanistic, and clinical evidence at the interface between CHIP and cancer immunotherapy using a three-level hierarchy: established inflammatory biology, suspected effects on antitumor immunity, and unresolved direct human causality. We also map the evidence by driver gene, clone size, tumor type, treatment modality, and detection context. Immunotherapy-specific data are concentrated in TET2-mutant CHIP; associations for DNMT3A and ASXL1 are weaker, and direct JAK2 data are absent. Clinical ICI cohorts report favorable TET2-associated efficacy signals and no evidence that checkpoint blockade selects CH clones. Separate studies associate CHIP with ICI myocarditis and with severe cytokine release syndrome, prolonged hematologic toxicity, and TP53-mutant clonal expansion after CAR T-cell therapy. These findings remain observational and context dependent. Current evidence does not support CHIP-guided treatment selection. Prospective, standardized studies are needed to determine whether specific CHIP phenotypes predict efficacy, toxicity, or neither.

## Introduction

Immune checkpoint inhibitors (ICIs) that block cytotoxic T-lymphocyte-associated protein 4 (CTLA-4) or the programmed cell death-1 (PD-1)/PD-L1 axis have produced durable responses across a wide range of cancers and have fundamentally changed the treatment of advanced malignancies (1). Nevertheless, only a minority of patients derive long-term benefit, and immune-related adverse events (irAEs) remain a major source of morbidity (1, 2). Biomarker research has focused predominantly on tumor-intrinsic features, such as PD-L1 expression, tumor mutational burden, and microsatellite instability, but host-level factors - age, systemic inflammation, immune competence, and comorbidities - are increasingly recognized as important determinants of both efficacy and toxicity (2).

Clonal hematopoiesis of indeterminate potential (CHIP) is the age-related clonal expansion of hematopoietic stem cells harboring somatic mutations in leukemia-associated genes, most commonly *DNMT3A*, *TET2*, and *ASXL1*, in the absence of cytopenia or dysplasia (3–5). Originally described through population-scale sequencing studies (4–7), CHIP is now recognized as one of the most common aging-related somatic states in humans, detectable in roughly 10% of individuals older than 65 years at conventional sequencing depths and in the large majority of middle-aged adults when ultra-sensitive error-corrected methods are used (5, 8, 9). Germline genetic variation shapes susceptibility to CHIP, and CHIP itself has been linked, through longitudinal and Mendelian randomization (MR) analyses, to an increased risk of hematologic malignancies and several solid cancers, including lung cancer (10, 11). CHIP has also been associated with a range of non-hematologic, age-related conditions, positioning it as a multisystem inflammatory state (42).

Two features make CHIP particularly relevant to cancer immunotherapy. First, CHIP is a driver of chronic, low-grade systemic inflammation - often framed as a component of “inflammaging” - through activation of the NLRP3 inflammasome, IL-1β, and IL-6 signaling (12–16). Second, CHIP is common in patients with cancer: it is detectable in approximately 25% of patients with non-hematologic malignancies in large sequencing cohorts (17) and in 26.5% of patients undergoing tumor-only clinical genomic profiling (18). Because CHIP mutations are frequently misattributed to tumor tissue in tumor-only sequencing and appear as incidental findings in cell-free DNA (cfDNA) assays, oncologists now encounter CHIP routinely in clinical practice (18, 19).

Emerging evidence suggests that CHIP may be more than an incidental finding: it may act as a host-intrinsic modifier of antitumor immunity and of the response to T-cell-based immunotherapies. Experimental studies demonstrate that TET2-mutant hematopoietic clones can reshape the tumor immune microenvironment, and clinical cohorts report improved outcomes with immune checkpoint inhibitor therapy (ICI) in patients with TET2-mutant CHIP (20, 21). In parallel, CHIP has been associated with an increased risk of ICI-related myocarditis and with severe cytokine release syndrome (CRS) and prolonged hematologic toxicity after chimeric antigen receptor (CAR) T-cell therapy (22–24). These observations are heterogeneous and, at face value, paradoxical: the same host state may enhance efficacy in one setting and aggravate toxicity in another.

Recent reviews have examined CH across cancer therapies (38), summarized its biology in myeloid and solid tumors (37), addressed incidental detection and clinical management in non-hematologic cancers (35), or focused on a single tumor type such as colorectal cancer (40). The present review has a narrower purpose: to evaluate CHIP in ICI and cellular immunotherapy using an explicit evidence hierarchy and a phenotype-specific map of efficacy and toxicity. This structure separates findings established in non-cancer models from immunotherapy-specific associations that remain unvalidated, and it makes the limits of inference visible for each driver gene and treatment context.

## Evidence identification and synthesis

This article is a critical narrative review, not a systematic review. We conducted targeted, non-systematic searches of PubMed/MEDLINE from database inception through August 2026, supplemented by cross-index checks in Crossref, OpenAlex, and Semantic Scholar and by screening reference lists of key reviews and primary studies. Search terms combined clonal hematopoiesis, CHIP, and specific driver genes with immunotherapy, immune checkpoint inhibitors, chimeric antigen receptor T cells, tumor-infiltrating lymphocytes, inflammation, immune-related adverse events, liquid biopsy, and Mendelian randomization. We prioritized population-scale sequencing studies, systematic reviews and meta-analyses, clinical cohorts, mechanistic studies, and genetic or causal-inference studies directly relevant to the CHIP-immunotherapy interface. Because this was an interpretive synthesis, we did not perform a formal meta-analysis or risk-of-bias grading and do not claim systematic completeness.

## CHIP: definition, detection, and epidemiology in cancer

### Definition and biology

CHIP was formally defined as the presence of a leukemia-associated somatic mutation (most commonly in *DNMT3A*, *TET2*, *ASXL1*, *JAK2*, *PPM1D*, or *TP53*) in peripheral blood cells at a variant allele frequency (VAF) of at least 2%, in individuals without cytopenia or dysplasia and without a diagnosis of a hematologic neoplasm (3). The concept emerged from whole-exome and targeted sequencing studies that identified age-related somatic mutations and clonal expansion in the blood of ostensibly healthy individuals, with an exponential increase in prevalence with age: 9.5% of persons aged 70–79 years in one population cohort (4), 10% of persons older than 65 years in another (5), and an increasing burden in the very old (4, 9). At ultra-sensitive sequencing depths, clonal hematopoiesis harboring myeloid-leukemia-associated mutations is detectable in 95% of healthy adults aged 50–60 years, indicating that CHIP represents the visible tip of a nearly universal aging phenomenon (8). Smoking, telomere length, and germline variation at loci such as *TET2* influence CHIP development and clonal burden (9–11).

CHIP is not a single entity. Different driver mutations confer different clonal dynamics and different systemic consequences: mutations in DNA damage response genes such as *TP53* and *PPM1D* expand under genotoxic selective pressure (25); *DNMT3A* mutations are the most common and, when present in isolation, are not associated with adverse clinical outcomes in meta-analyses (26); and *TET2* mutations are distinguished by their strong inflammatory signature (13–15). These gene-specific differences are central to interpreting the immunotherapy literature and are discussed further below.

### CHIP in patients with cancer

CHIP is more prevalent in patients with cancer than in age-matched general populations. In a deep, targeted sequencing analysis of paired tumor and blood samples from 8,810 patients with non-hematologic malignancies, clonal hematopoiesis (CH) was identified in 25% of patients, with 4.5% harboring presumptive leukemia driver mutations; CH was associated with age, prior radiation therapy, and tobacco use, and mutations in *PPM1D* and *TP53* were enriched after prior chemotherapy (17). CHIP was associated with an increased incidence of subsequent hematologic cancers, and leukemia-driver CH was associated with shorter overall survival (17). In a larger tumor-only sequencing cohort of 17,469 patients, CH-associated mutations were present in the blood of 26.5% of patients, and 5.2% of patients would have had at least one CH mutation erroneously attributed to the tumor in the absence of matched blood sequencing; nearly half of these variants were annotated as oncogenic or likely oncogenic (18). High-intensity sequencing of cfDNA similarly identified clonal hematopoiesis as a major source of non-tumor plasma variants, underscoring the need for paired controls in liquid biopsy workflows (19). A large Chinese pan-cancer cohort using paired tumor-blood sequencing reported CH in 28.6% of patients, with CH-associated variants frequently detectable in matched tumor tissue, providing evidence of tumor-infiltrating clonal hematopoiesis in clinical populations (27). In cfDNA-based cohorts, CHIP prevalence ranges from approximately 10% to 30%, increasing with age (28), and in a recent real-world cohort of 2,040 patients with advanced solid tumors, CHIP was detected in 25.7% (29).

### Therapy-related clonal hematopoiesis and clonal dynamics

Cancer therapies themselves shape the fitness landscape of clonal hematopoiesis. Chemotherapy, radiation, and other genotoxic exposures select for mutations in DNA damage response genes - *TP53, PPM1D*, and *CHEK2* - and sequential sampling demonstrates that these clones outcompete other clones under treatment pressure (25). *ASXL1* mutations are enriched in current or former smokers (25). These dynamics are clinically important because high-risk CHIP clones can expand to therapy-related myeloid neoplasms (tMNs), and the increasing implementation of clinical sequencing at diagnosis provides an opportunity to identify at-risk patients prospectively (25). For immunotherapy specifically, available data indicate that immune checkpoint blockade does not exert comparable clonal selection pressure: in paired blood samples from 91 patients with melanoma or basal cell carcinoma treated with ICIs, clonal hematopoiesis burden and composition were highly stable over time, with no expansion of *TP53-* or *PPM1D-mutant* clones (30). Thus, whereas genotoxic therapies promote CH evolution, checkpoint blockade appears to be a neutral force in CH selection, an important distinction when interpreting CHIP-immunotherapy associations (30). Beyond genotoxic agents, non-genotoxic cancer therapies also shape CHIP dynamics. Targeted therapies that perturb the hematopoietic niche or systemic immune milieu, including tyrosine kinase inhibitors and endocrine therapies, may modulate clone fitness, and the effect of treatment on CHIP must therefore be interpreted by drug class rather than by treatment categorization alone (25). Lymphodepleting conditioning regimens used before chimeric antigen receptor T-cell therapy create a distinct selective and inflammatory environment in which CH clones, particularly TP53-mutant clones, can expand toward therapy-related myeloid neoplasia and predispose to prolonged cytopenias (24). In addition, the relationship between primary cancer type and CHIP is not uniform: the frequency, driver spectrum, and clinical consequence of CH differ between hematologic and solid malignancies and among individual solid tumors, reflecting differences in prior genotoxic exposure, smoking, and tumor-specific surveillance (17).

## From clonal hematopoiesis to immune dysregulation: a three-level mechanistic framework

The mechanistic evidence linking CHIP to immunotherapy outcomes can be organized into three levels: established inflammatory biology in experimental models (Level 1), biologically plausible but incompletely validated effects on antitumor immunity (Level 2), and unresolved direct human causality together with the sources of heterogeneity that limit inference (Level 3).

### Level 1: established biology, CHIP as a driver of systemic inflammation

Experimental studies establish a causal link between CHIP and inflammatory signaling, although the strongest evidence comes from non-cancer cardiovascular models: Tet2-deficient hematopoiesis increases macrophage NLRP3/IL-1beta activity, Tet2 loss impairs Hdac2-mediated repression of IL-6, and Dnmt3a loss produces a distinct cytokine program (15, 16, 31). Human cardiovascular and genetic studies support gene- and clone-size-dependent inflammatory effects (14, 32), but here these findings serve only as a mechanistic anchor rather than direct evidence for cancer immunotherapy outcomes. With respect to the IL-6 pathway, the genetic evidence for targetability derives from a Mendelian-randomization design: Bick and colleagues studied the naturally occurring germline loss-of-function variant IL6R p.Asp358Ala as a genetic proxy for reduced IL-6 signaling and observed lower cardiovascular risk among carriers of large CHIP clones (32). This genetic-proxy evidence is not equivalent to pharmacologic IL-6 pathway blockade (for example, tocilizumab or siltuximab) and should not be interpreted as demonstrating efficacy of anti-IL-6 therapy in humans.

Cancer-specific data establish the clinical relevance of CH more directly. In oncology cohorts, CH is present in about one-quarter of patients with non-hematologic malignancies and is associated with subsequent hematologic cancer risk; leukemia-driver CH is also associated with shorter overall survival (17). Tumor-only sequencing can misattribute blood-derived variants to the tumor, whereas paired tumor-blood studies show that CH-derived immune cells can be detected within tumor tissue (18, 27). Across 88 studies, CHIP was associated with all-cause mortality, cancer mortality, lung cancer, and hematologic malignancy, with effect estimates modified by driver gene, clone size, and patient characteristics (26). These observations support relevance in cancer populations but do not establish an immunotherapy-specific causal effect.

### Level 2: suspected biology, CHIP as a modifier of antitumor immunity

Evidence that CHIP modifies antitumor immunity is strongest for TET2. In solid-tumor mouse models, Tet2-mutant myeloid cells accumulated in tumors, improved macrophage antigen presentation and CD8+ T-cell cross-priming, and enhanced response to ICI (20). Independent models showed that the treatment effect required phagocytes, CD4+ T cells, and CD8+ T cells and was lost when only 20% of hematopoiesis was Tet2 mutant (21). Single-cell analyses linked Tet2-mutant monocytes to costimulatory programs and Tet2-mutant T cells to stronger memory signatures with less exhaustion and regulatory differentiation (21). TET2 loss also promotes CD8+ T-cell memory differentiation (33). A clinical case of vector-mediated TET2 disruption in an infused CAR T-cell clone produced durable remission (34), but this engineered event is not equivalent to host TET2-mutant CHIP. Notably, in this index case the vector-disrupted TET2 clone expanded to account for more than 94% of active CAR T cells at peak response, without evidence of neoplastic transformation, underscoring the potency-enhancing potential of TET2 loss *in vivo* (34).

Human data are correlative. Colorectal cancer and melanoma tumors from patients with TET2-mutant CH showed greater immune infiltration, inflammation, and T-cell activation (21), and large NSCLC and colorectal cancer cohorts associated TET2-mutant CH with better outcomes specifically after ICI (20). These concordant preclinical and clinical observations support suspected biology, but they do not establish a causal pathway in patients.

### Level 3: unresolved human causality and sources of heterogeneity

No human study in the reviewed literature demonstrates a complete causal chain from a CHIP clone to altered tumor immunity and then to an immunotherapy outcome. Nearly all clinical findings are retrospective or observational, and no prospective trial has validated CHIP-stratified treatment effects. The divergent results are therefore best interpreted through the heterogeneity of CHIP phenotypes and study designs rather than as a single CHIP-positive effect. **Figure 1** conceptual framework linking clonal hematopoiesis of indeterminate potential to cancer immunotherapy outcomes. CHIP arises from age-related clonal expansion of hematopoietic stem cells harboring driver mutations (*TET2, DNMT3A, ASXL1, TP53, PPM1D*, and others), with clone size and clonal dynamics shaping systemic effects. CHIP promotes chronic low-grade inflammation (NLRP3/IL-1b and IL-6 signaling) and can alter antitumor immunity, particularly through TET2-mutant myeloid antigen presentation and memory-like T-cell programs. These host states may modulate both the efficacy (immune checkpoint therapy, CAR T cells) and the toxicity (immune checkpoint inhibitor myocarditis, cytokine release syndrome, cytopenias, therapy-related myeloid neoplasms) of cancer immunotherapy. Contextual modifiers - age, prior genotoxic therapy, tumor type, and detection method - and dashed causal arrows denote that the direction, magnitude, and causality of these links remain incompletely established. CAR, chimeric antigen receptor; IL, interleukin; NLRP3, NOD-, LRR- and pyrin domain-containing protein 3.

**Figure 1:**
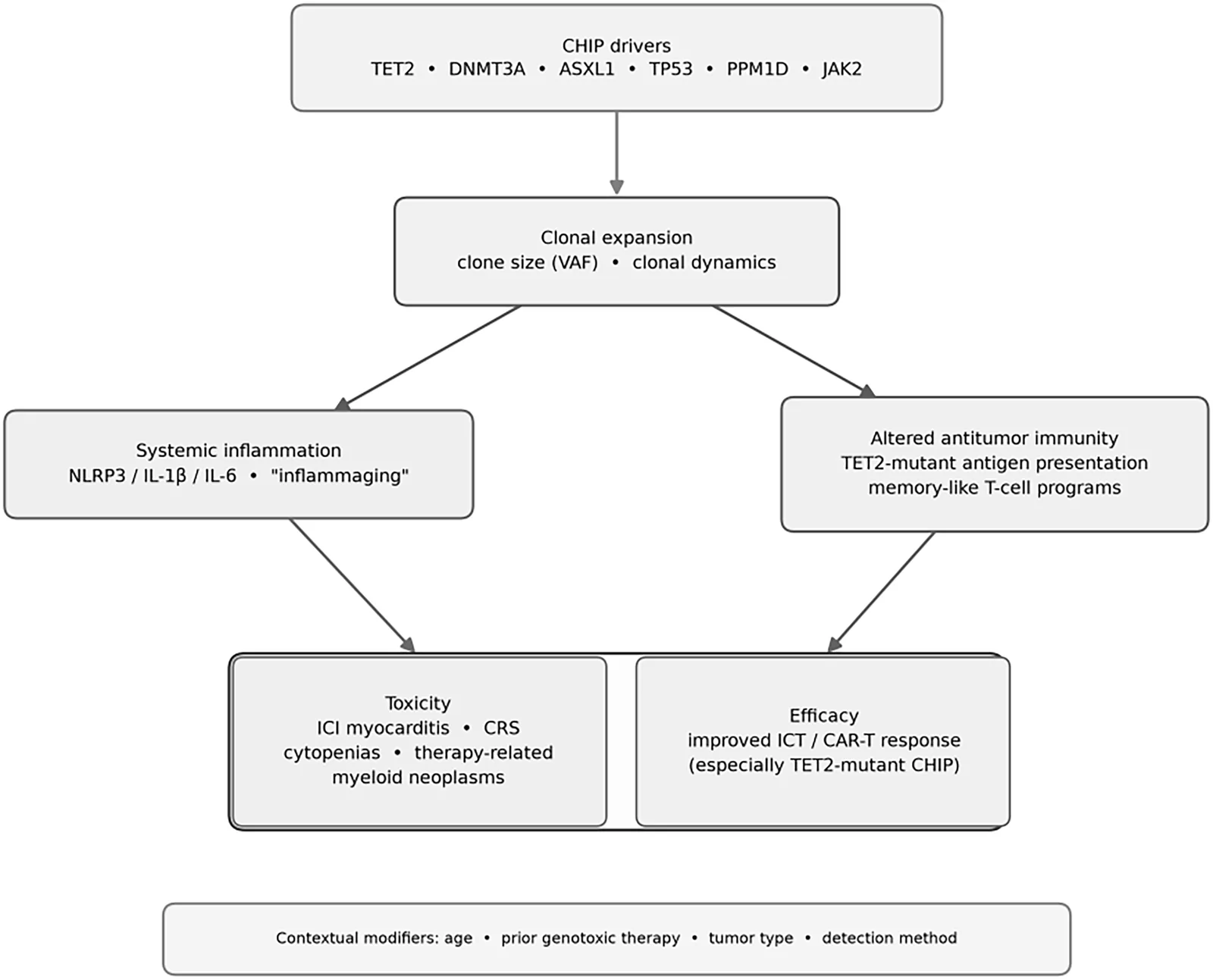
CHIP as a host-intrinsic modifier of cancer immunotherapy. CHIP arises from age-related clonal expansion of hematopoietic stem cells harboring driver mutations (TET2, DNMT3A, ASXL1, TP53, PPM1D, and others), with clone size and clonal dynamics shaping systemic effects. CHIP promotes chronic low-grade inflammation (NLRP3/IL-1β and IL-6 signaling) and can alter antitumor immunity, particularly through TET2-mutant myeloid antigen presentation and memory-like T-cell programs. These host states may modulate both the efficacy (immune checkpoint therapy, CAR T cells) and the toxicity (immune checkpoint inhibitor myocarditis, cytokine release syndrome, cytopenias, therapy-related myeloid neoplasms) of cancer immunotherapy. Contextual modifiers - age, prior genotoxic therapy, tumor type, and detection method - and dashed causal arrows denote that the direction, magnitude, and causality of these links remain incompletely established. CAR, chimeric antigen receptor; IL, interleukin; NLRP3, NOD-, LRR- and pyrin domain-containing protein 3.

#### Driver gene and clone size

TET2-mutant CHIP is the best-characterized immunotherapy phenotype (20, 21, 29). DNMT3A-mutant CHIP is more prevalent, but its inflammatory program differs from TET2 in experimental hematopoiesis (31), isolated DNMT3A CHIP was not associated with adverse outcomes in a population meta-analysis (26), and the ICI-specific clinical signal was non-significant (29). ASXL1 findings are observational and inconsistent (25, 29). For JAK2-mutant CHIP, the cited human data establish an association with cardiovascular events (14), but we identified no study linking JAK2-mutant CHIP to tumor immune states or ICI/CAR-T outcomes. JAK2 is therefore a named evidence gap rather than a basis for mechanistic extrapolation. TP53 and PPM1D require separate interpretation because genotoxic therapy selects these clones (25), and TP53-mutant CH can expand after CAR T-cell therapy (24). Clone size may also matter: larger clones carry greater disease risk in non-immunotherapy studies (32, 39), whereas ICI studies use different VAF thresholds and have not established a dose-response relationship (28–30). Whether any host VAF threshold or clonal burden has been shown to alter antitumor immunity or toxicity remains unresolved. CHIP-definition thresholds vary markedly across the cited studies, including VAF ≥2% in the ICI cohorts (28, 29), VAF >1% in the clonal-stability cohort (30), and large-clone (VAF >10%) strata in the cardiovascular-risk cohorts (14, 32), and no study has demonstrated a dose-response relationship between host clonal burden and immunotherapy efficacy or toxicity. The apparent threshold effect in the transgenic mouse model, in which the treatment benefit was lost when only 20% of hematopoiesis was mutant (21), provides a rationale for defining such thresholds, but this remains untested in patients.

#### Tumor type and treatment context

Treatment associations vary by tumor and modality. TET2-specific favorable ICI signals have been reported in NSCLC, colorectal cancer, and melanoma (20, 21). In metastatic pancreatic adenocarcinoma, aggregate DNMT3A/TET2/ASXL1 CHIP was associated with a non-significant PFS trend in the ICI arm, while a mixed-tumor chemotherapy cohort showed a non-significant trend in the opposite direction (28). These estimates cannot be generalized across tumors because immunogenicity, myeloid infiltration, treatment combinations, and endpoints differ. CAR T-cell studies address hematologic malignancies and a distinct setting shaped by lymphodepletion, cytokine exposure, and clonal selection (23, 24).

#### Detection context and methodological limitations

Study design adds another layer of heterogeneity. Definitions vary in gene-panel content, VAF threshold, sequencing platform, and sampling time (28–30). Tumor-only sequencing can misattribute CH variants to the tumor, cfDNA assays readily detect blood-derived variants, and tumor-infiltrating CH complicates tissue interpretation (18, 19, 27). Paired blood or error-corrected sequencing and transparent reporting of genes, VAF, and clonal burden are needed for comparable studies (36).

Most outcome studies are retrospective, registry-based, or drawn from surveillance-enriched cohorts. Age, smoking, obesity, infection, prior genotoxic therapy, comorbidity, and baseline inflammation can affect both CHIP detection and treatment outcomes. The myocarditis cohort illustrates this problem: its high event rate and cardio-oncology surveillance limit generalizability despite prospective ascertainment (22). Gene-specific subgroup sizes are small, endpoints differ, and no randomized or causal-inference study has tested CHIP-guided treatment decisions. A further, underappreciated limitation is heterogeneity in the timing of blood sampling across published retrospective studies. In many of the cited cohorts, the blood used for CHIP sequencing was collected at variable timepoints, frequently after multiple lines of cytotoxic therapy or even during active ICI treatment, rather than consistently at baseline before ICI initiation. Because treatment itself can alter clone burden and systemic inflammation, the absence of standardized pretreatment sampling makes it difficult to disentangle baseline CHIP from treatment-induced clonal dynamics and strengthens the case for prospective, time-standardized biobanking.

#### Causal interpretation

At least four explanations remain compatible with the clinical data: CHIP may modify antitumor immunity; CHIP and treatment outcome may share upstream aging or inflammatory causes; prior therapy may alter clonal fitness; and detection or surveillance may create an apparent association. Existing Mendelian-randomization studies address lung cancer risk and CHIP susceptibility, not immunotherapy-specific outcomes (11, 39, 43). Prospective cohorts with serial clonal and immune profiling, prespecified gene- and clone-size analyses, and treatment-specific causal designs are required to distinguish these models.

## CHIP and immune checkpoint inhibitors: clinical evidence

### Efficacy signals

Clinical data, although heterogeneous, point toward a genotype-specific association between CHIP and ICI efficacy, with the most consistent signal for *TET2*-mutant CHIP.

In the Canadian Cancer Trials Group PA.7 trial of gemcitabine and nab-paclitaxel with or without durvalumab plus tremelimumab in metastatic pancreatic adenocarcinoma, patients with CHIP (defined as *DNMT3A, TET2*, or *ASXL1* mutations at VAF ≥2% in cfDNA) who received immunotherapy showed a non-significant association with improved progression-free survival compared with CHIP-negative patients (HR 0.55; p = 0.079), whereas in the chemotherapy-treated PREDiCT-l cohort, CHIP-positive patients showed a non-significant trend toward worse progression-free survival (HR 1.82; p = 0.059); these estimates come from an integrated analysis of 465 patients across color the PA.7 (pancreatic), CO.26 (colorectal), and PREDiCT-l (pan-solid-tumor) cohorts (28). In a real-world analysis of 2,040 patients with advanced solid tumors treated with ICIs, CHIP was detected in 25.7% of patients and was associated with significantly prolonged time to treatment discontinuation (median 10.1 vs. 7.9 months; HR 0.81; p = 0.005); *TET2* mutations were independently associated with longer treatment duration (HR 0.77; p = 0.035), whereas *DNMT3A* showed a similar but non-significant trend and *ASXL1-positive* patients had a lower overall response rate despite a trend toward longer treatment duration (29). In large clinico-genomic cohorts of 35,971 patients with non-small cell lung cancer and 25,064 patients with colorectal adenocarcinoma, *TET2-mutant* CHIP was associated with improved outcomes specifically with ICI, and not with other treatment modalities (20). In patients with melanoma treated with ICIs, *TET2-mutant* CHIP was associated with approximately six-fold greater odds of clinical benefit (21).

Several caveats apply. Effect sizes are modest, confidence intervals are wide in the smaller cohorts, definitions of CHIP vary, and no study has yet established whether the association is driven by *TET2* mutation per se, by clone size, or by an underlying inflammatory state for which *TET2*-mutant CHIP is a marker. The observation that the efficacy benefit in mice was lost when only 20% of hematopoiesis was mutant suggests a clone-burden threshold that has not yet been defined in human cohorts (21). In addition, the endpoints used to define clinical benefit varied across studies. Residual confounding by baseline lifestyle and tumor characteristics is an additional concern. Smoking promotes clonal expansion of TET2-deficient hematopoietic cells (44), and smoking also increases tumor mutational burden (TMB), an established independent predictor of ICI efficacy (2). The cited retrospective cohorts (20, 21, 28, 29) did not uniformly report adjustment for both smoking status and baseline TMB in their gene-specific analyses, so we cannot exclude the possibility that the TET2-associated efficacy signal partly reflects a higher TMB among TET2-mutant patients rather than an effect of TET2-mutant hematopoiesis itself. Dedicated smoking- and TMB-adjusted analyses are required before TET2-mutant CHIP can be interpreted as an independent predictive biomarker.

### Neutral effects on clonal selection

Unlike cytotoxic therapy, immune checkpoint blockade does not appear to select for or expand CHIP clones. In paired pre- and post-treatment blood samples from 91 patients treated with ICIs, 41% had clonal hematopoiesis at VAF greater than 1%, and 87 of 88 CH mutations were present in both samples with a high correlation of VAFs (R² = 0.95), indicating clonal stability during ICI exposure (30). No expansion of *TP53*- or *PPM1D*-mutant clones was observed (30). These findings separate the question of whether CHIP modifies ICI efficacy from the question of whether ICIs alter CH evolution, and they suggest that ICI-related hematologic sequelae are unlikely to be driven by clonal selection in the same way as after chemotherapy.

### Toxicity signals

CHIP has been associated with organ-specific inflammatory toxicity during ICI therapy. In a prospective cardio-oncology cohort of 88 patients with solid tumors receiving ICIs, 50% harbored CHIP variants; the cumulative incidence of ICI myocarditis was significantly higher in CHIP-positive than CHIP-negative patients (73% vs. 41%; p = 0.003), and in multivariable competing-risk analysis, CHIP was associated with a more than twofold higher risk of ICI myocarditis (adjusted HR 2.74), comparable to the risk associated with dual ICI therapy (22). CHIP-positive patients also had higher all-cause mortality in this cohort (22). In the larger ICI cohorts discussed above, no significant association was found between CHIP and the overall incidence of grade ≥3 irAEs (28, 29), suggesting that CHIP may preferentially modulate specific inflammatory pathways - such as cardiac inflammation - rather than global irAE burden. The generalizability of this finding is limited because Chehayeb and colleagues evaluated a highly selected cardio-oncology referral cohort in which the overall incidence of ICI myocarditis was extraordinarily high at 57%, far above the less than 1% incidence reported in unselected, real-world ICI-treated populations. Regarding non-cardiac immune-related adverse events, the PA.7/PREDiCT-l integrated analysis (28) reviewed grade ≥3 adverse events and dose-limiting toxicities and found no difference by CHIP status, but it did not separately report organ-specific non-cardiac irAEs such as colitis, pneumonitis, or endocrinopathies; the larger real-world ICI cohort likewise reported no association with overall grade ≥3 irAEs (29). Thus, while CHIP is associated with an apparent excess of myocarditis in surveillance-enriched cohorts, no association with non-cardiac irAEs has been established.

### Summary of clinical studies

**Table 1** summarizes the principal clinical studies of CHIP and immune checkpoint inhibitor outcomes.

**Table 1:** Clinical studies of CHIP and immune checkpoint inhibitor outcomes.

Tumor type(s)/study	Study level	Population and design	CHIP definition	Efficacy or clonal finding	Toxicity finding
Colorectal cancer and melanoma Rondeau et al., 2026 (21)	Both	Mouse models; human colorectal cancer and melanoma tumors; melanoma ICI cohort	Hematopoietic TET2 inactivation/TET2-mutant CH	Enhanced ICI response in mice; TET2-mutant tumors showed immune activation; approximately six-fold greater odds of clinical benefit in melanoma	Not reported
Melanoma and basal cell carcinoma Miller et al., 2020 (30)	Clinical	91 ICI-treated patients; paired blood before and after treatment	CH at VAF >1%; targeted panel	No efficacy endpoint; 87/88 CH mutations persisted and VAFs were highly correlated (R2 = 0.95), with no clone expansion	No TP53/PPM1D selection; no hematologic toxicity signal reported
Metastatic pancreatic adenocarcinoma, metastatic colorectal cancer, and mixed solid tumors Krishnan et al., 2025 (28)	Clinical	465 patients in PA.7, CO.26, and PREDiCT-l cohorts	DNMT3A/TET2/ASXL1 at VAF >=2% in cfDNA	PA.7 immunotherapy arm: PFS HR 0.55 (95% CI 0.28-1.07; p = 0.079) for CHIP+ vs. CHIP-; PREDiCT-l chemotherapy: PFS HR 1.82 (95% CI 0.98-3.38; p = 0.059)	No difference in grade >=3 or dose-limiting adverse events with immunotherapy or chemotherapy
Mixed solid tumors under cardio-oncology surveillance Jaber Chehayeb et al., 2024 (22)	Clinical	88 ICI-treated patients; prospective cardio-oncology cohort	CHIP by whole-exome sequencing	Not evaluated	ICI myocarditis: 73% vs. 41% (p = 0.003); adjusted HR 2.74
NSCLC and colorectal adenocarcinoma Herbrich et al., 2026 (20)	Both	35,971 NSCLC and 25,064 colorectal cancer patients in clinico-genomic cohorts; mouse models	TET2-mutant CH	Improved outcomes specifically with ICI in both human cohorts; enhanced ICI response in mice	Not reported
Pan-solid tumors Fujita et al., 2026 (29)	Clinical	2,040 patients with advanced solid tumors; real-world liquid biopsy and ICI data	DNMT3A/TET2/ASXL1 at VAF >=2%	Longer TTD for CHIP+ vs. CHIP- (10.1 vs. 7.9 months; HR 0.81); TET2 HR 0.77; DNMT3A trend was non-significant; ASXL1 was associated with lower ORR and a trend toward longer TTD	No association with grade >=3 irAEs

CH, clonal hematopoiesis; CHIP, clonal hematopoiesis of indeterminate potential; HR, hazard ratio; ICI, immune checkpoint inhibitor; ORR, overall response rate; PFS, progression-free survival; TTD, time to treatment discontinuation; VAF, variant allele frequency.

## CHIP and cellular immunotherapies

### CAR T-cell therapy

CHIP is common in patients with B-cell malignancies and multiple myeloma before CAR T-cell therapy, and emerging data associate it with both efficacy-relevant and toxicity-relevant effects. In a cohort of 62 patients with non-Hodgkin lymphoma or multiple myeloma treated with CD19- or BCMA-directed CAR T cells, 24% had at least one pathogenic CH mutation before infusion; CH was associated with a significantly higher incidence of grade ≥2 CRS (60% vs. 28%; odds ratio [OR] 3.9; p = 0.027) (23). In a larger cohort of 213 patients with myeloma treated with BCMA-directed CAR T cells, persistent high-grade hematologic toxicity at day 100 occurred in 19% of patients and was associated with shorter progression-free and overall survival; the combination of baseline CH and elevated ferritin was strongly associated with delayed hematologic recovery (adjusted HR 0.38 for recovery; p = 0.006), and 9% of patients developed secondary myeloid disease, including high-grade myelodysplastic syndrome in 5% (24). Notably, TP53-mutated CH clones expanded dramatically after CAR T-cell therapy (median VAF from 3.4% to 44.0% at myeloid disease diagnosis), and 67% of patients with baseline TP53-mutated CH developed myelodysplastic syndrome, whereas non-TP53 CH did not show comparable expansion (24). These data indicate that CHIP - particularly TP53-mutant CHIP - may identify patients at high risk of CAR-T-associated hematologic toxicity and secondary myeloid neoplasia.

The relationship between *TET2* function and CAR T-cell efficacy deserves particular mention. In the index clinical case of *TET2*-disrupted CAR T cells, disruption of *TET2* by vector integration promoted clonal expansion, central memory differentiation, and durable remission in chronic lymphocytic leukemia (34). This case, together with the experimental demonstration that *TET2* loss promotes CD8+ T-cell memory differentiation (33), provides a strong biological rationale that *TET2*-mutant host hematopoiesis could enhance T-cell-based therapies. However, it is important to distinguish engineered *TET2* disruption within the infused CAR T-cell product from pre-existing *TET2*-mutant CHIP in the host; the latter may influence the systemic immune milieu rather than the intrinsic fitness of the infused cells.

### Other cellular therapies

Data on CHIP and other cellular therapies, including tumor-infiltrating lymphocyte therapy, remain limited, and dedicated studies are needed. The broader interplay between clonal hematopoiesis and diverse cancer therapeutic platforms has been reviewed comprehensively elsewhere (38). Current knowledge is therefore extrapolated from the ICI and CAR-T literature, with the caveat that conditioning regimens, cytokine exposure, and clonal selection pressures differ substantially across cellular platforms.

## Clinical implications by tumor type and treatment setting

For NSCLC and colorectal cancer, the most relevant evidence is the TET2-specific association with better ICI outcomes in large clinico-genomic cohorts (20). In melanoma, TET2-mutant CH was associated with clinical benefit, supported by tumor and mouse data (21), while paired sampling in melanoma and basal cell carcinoma showed stable CH clones during ICI exposure (30). These findings are not prospective biomarker validations and should not determine ICI eligibility.

In metastatic pancreatic adenocarcinoma, aggregate DNMT3A/TET2/ASXL1 CHIP was associated with a non-significant PFS trend in an ICI-containing arm; the same study found an opposite non-significant trend in a mixed-tumor chemotherapy cohort (28). Pan-solid real-world data showed longer ICI treatment duration overall, with the clearest gene-specific signal for TET2 and weaker or mixed findings for DNMT3A and ASXL1 (29). These results justify prespecified stratification in future trials, not cross-tumor extrapolation.

Toxicity evidence should be applied by treatment setting. The ICI-myocarditis association arose in a surveillance-enriched cardio-oncology cohort and requires replication before CHIP-directed cardiac monitoring becomes standard (22). In CAR T-cell therapy, baseline CH was associated with severe CRS, and CH combined with inflammation predicted delayed hematologic recovery; TP53-mutant clones carried the clearest signal for expansion toward myeloid disease (23, 24). These data support prospective hematologic follow-up studies but do not establish a universal monitoring protocol.

## Phenotype-specific evidence map

**Table 2** consolidates the phenotype-specific evidence map and shows where the literature is concentrated or absent.

**Table 2:** Phenotype-specific evidence map for CHIP in immunotherapy.

Dimension	Phenotype	Mechanistic basis	Clinical direction in immunotherapy	Certainty
Driver gene	*TET2*	Inflammatory myelopoiesis; enhanced antigen presentation and CD8+ cross-priming; memory-like T-cell programs (15, 20, 21, 31, 33)	Improved ICI outcomes across NSCLC, CRC, melanoma, and pan-cancer cohorts (20, 21, 29)	Moderate (concordant mouse + human data, but observational)
Driver gene	*DNMT3A*	Most common CHIP driver; Dnmt3a loss produces a cytokine profile distinct from Tet2 loss (31)	Non-significant trend toward longer ICI treatment duration; no validated gene-specific efficacy or toxicity effect (29)	Low
Driver gene	*ASXL1*	Enriched in smokers (25)	Lower ORR but longer treatment duration trend (29)	Low
Driver gene	JAK2	Gene-specific cardiovascular association reported, but no immunotherapy-specific mechanism established in the cited literature (14)	No ICI- or CAR-T-specific outcome data identified	Low (named evidence gap)
Driver gene	*TP53*/*PPM1D*	DNA damage response clones; selected by genotoxic therapy; high tMN risk (25)	No ICI-specific efficacy data; *TP53*-CH expansion and high MDS risk after CAR-T (24)	Low-moderate (toxicity)
Clone size	VAF ≥10%	Larger clones carry greater inflammatory and disease risk (32, 39)	ICI-specific VAF threshold not established; trials used VAF ≥2% (28, 29)	Low
Treatment context	ICI	Host inflammatory-immune state may enhance antitumor immunity	Favorable efficacy signal, organ-specific toxicity (myocarditis) (20–22, 29)	Moderate
Treatment context	CAR-T	Myeloid activation, conditioning, and cytokine storm engage CH clones; *TP53* clones expand (23, 24)	Toxicity-dominant: CRS, cytopenias, secondary myeloid neoplasia (23, 24)	Moderate
Treatment context	Chemotherapy	Genotoxic selection of *TP53*/*PPM1D* clones (25)	Trend toward worse PFS in CHIP+ patients (28)	Low
Detection context	Tumor-only/cfDNA	CH mutations misattributed to tumor; cfDNA contamination (18, 19)	Confounding of tumor genotyping and biomarker interpretation	High (established)

Certainty reflects the strength and concordance of the available evidence: High, replicated or mechanistically established; Moderate, concordant mechanistic and observational data without prospective validation; Low, single or heterogeneous observational reports; Low-moderate, emerging but limited data. These labels correspond to the evidence hierarchy in the text, including Level 3 where direct human causality remains unresolved. CH, clonal hematopoiesis; CHIP, clonal hematopoiesis of indeterminate potential; CRC, colorectal cancer; ICI, immune checkpoint inhibitor; MDS, myelodysplastic syndrome; NSCLC, non-small cell lung cancer; ORR, overall response rate; PFS, progression-free survival; tMN, therapy-related myeloid neoplasm; VAF, variant allele frequency.

## Discussion

The cancer-focused evidence supports a genotype- and context-specific model rather than a uniform CHIP-positive state. TET2-mutant hematopoiesis can alter antigen presentation and leukocyte states in tumors, and the same genotype has the most consistent favorable ICI association in human cohorts (20, 21, 29). By contrast, direct immunotherapy data for DNMT3A and ASXL1 are limited, and JAK2-specific data are absent. Toxicity follows a different pattern: aggregate CHIP has been associated with myocarditis during ICI therapy, whereas CAR T-cell studies link CH, inflammation, and TP53-mutant clonal expansion to hematologic complications (22–24).

### Consolidated heterogeneity, confounding, and limitations

The main interpretive constraint is that phenotype and ascertainment are entangled. Driver gene, clone size, prior treatment, tumor type, assay, and sampling time all influence who is labeled CHIP positive and what outcome is observed. Retrospective designs and small gene-specific subgroups leave residual confounding by age, smoking, comorbidity, infection, and baseline inflammation. Current studies therefore support association, not treatment-selection utility. In routine practice, the immediate implication remains diagnostic: suspected blood-derived variants in tumor-only or cfDNA results should be confirmed when they could alter management (18, 19). **Figure 2** proposed framework for integrating CHIP assessment into cancer immunotherapy practice. Incidental CHIP detection during tumor-only genomic profiling or cell-free DNA sequencing should be confirmed with paired blood or error-corrected sequencing using validated curation standards, then classified by driver gene, variant allele frequency, and clonal dynamics. On current evidence, CHIP should not be used as a routine treatment-selection tool; rather, it may inform hypothesis-driven toxicity surveillance and stratify patients in prospective immunotherapy research. The pathway is intended as an interpretive framework for clinical research and does not replace existing oncology, cardio-oncology, or hematology guidelines.

**Figure 2:**
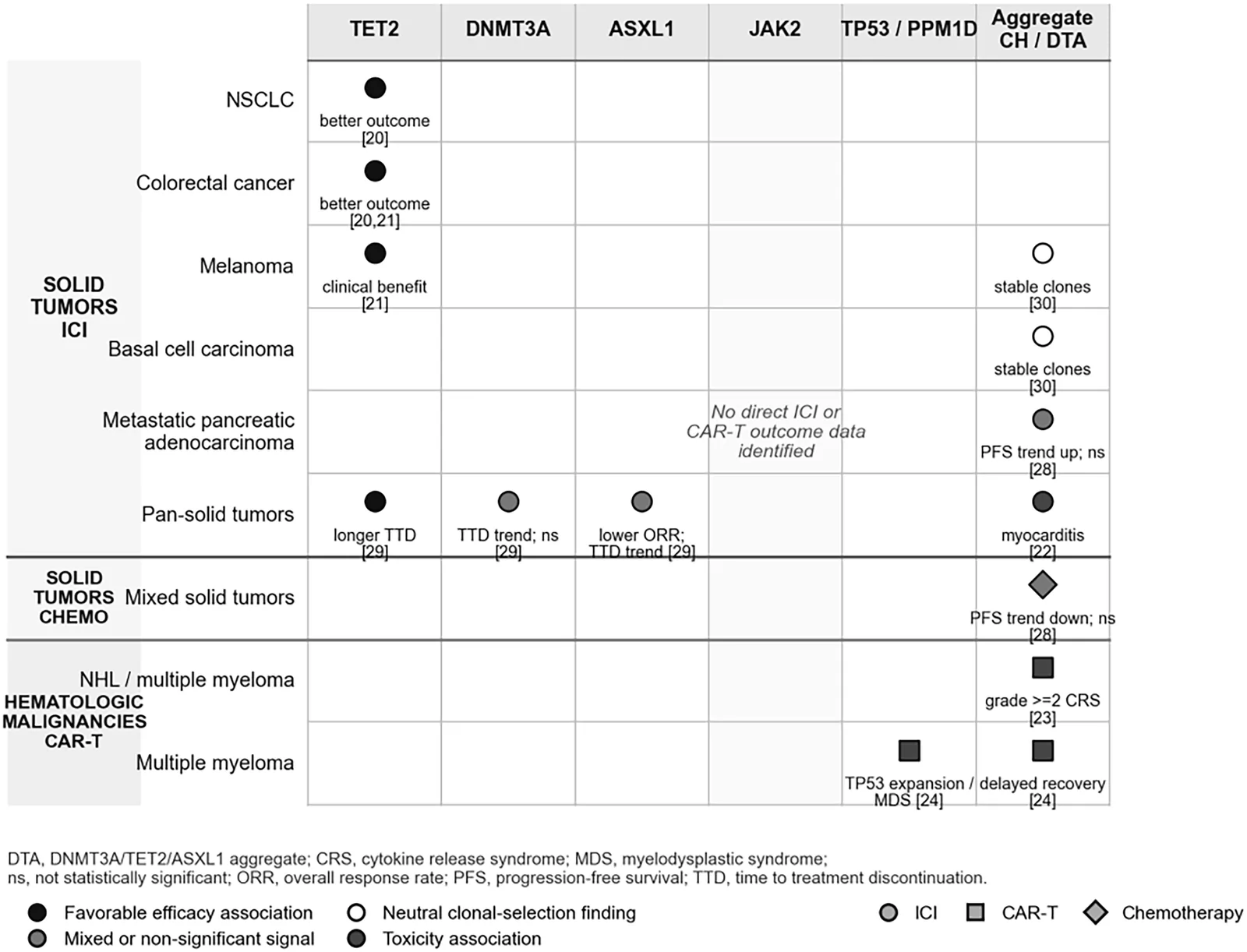
Proposed framework for integrating CHIP assessment into immunotherapy practice. Rows identify tumor or treatment settings, columns identify driver-gene phenotypes or aggregate CH/CHIP, and marker shape identifies treatment modality. A black marker denotes a favorable efficacy association, medium gray denotes a mixed or statistically non-significant signal, white denotes neutral clonal selection, and dark gray denotes a toxicity association. Empty cells indicate that no direct treatment-outcome evidence was identified in the studies reviewed. Aggregate DTA refers to studies that combined DNMT3A, TET2, and ASXL1 rather than reporting a gene-specific effect. The map displays associations, not causal effects, and separates solid-tumor ICI evidence from chemotherapy and hematologic CAR T-cell settings (20–24, 28–30). CH, clonal hematopoiesis; CRS, cytokine release syndrome; ICI, immune checkpoint inhibitor; MDS, myelodysplastic syndrome; NHL, non-Hodgkin lymphoma; NSCLC, non-small cell lung cancer; ORR, overall response rate; PFS, progression-free survival; TTD, time to treatment discontinuation.

### Research priorities

Future ICI and CAR T-cell cohorts should prespecify CHIP assessment, confirm variant origin with paired blood or error-corrected sequencing, report driver gene and VAF, and collect serial clonal and immune measurements (36). Analyses should be stratified by tumor type and treatment modality and should include treatment-specific causal designs. Mechanistic studies are especially needed for DNMT3A, ASXL1, and JAK2, for which direct immunotherapy evidence is sparse or absent. Intervention studies of IL-1beta or IL-6 pathways remain hypothesis driven: supportive inflammatory biology and the CANTOS lung-cancer signal do not establish benefit in CHIP-defined immunotherapy populations (11, 15, 32, 41). In the causal-inference framework, colocalization refers to analyses that test whether two traits, for example a CHIP-associated germline signal and an immunotherapy outcome, share causal genetic variants at the same locus, thereby supporting a shared causal mechanism rather than independent associations. Genetically informed cohort designs, such as Mendelian randomization that uses germline instruments for CHIP-associated phenotypes, estimate the effect of a heritable exposure on an outcome while exploiting the random assortment of alleles and are less susceptible to reverse causation and confounding than observational biomarker studies. Target trial emulation applies the design and analysis principles of a randomized trial to observational data. Applied to CHIP, these approaches would test whether genetically predicted CHIP-related inflammation causally affects immunotherapy efficacy and toxicity independent of the observational association.

## Conclusion

CHIP is common in patients with cancer, but immunotherapy evidence is uneven across genes and treatment settings. TET2-mutant CHIP has the most consistent favorable ICI association and the strongest mechanistic support (20, 21, 29). Checkpoint blockade itself did not expand CH clones in paired samples (30). Separate cohorts linked CHIP to ICI myocarditis and CAR-T-associated inflammatory and hematologic toxicity, including TP53-mutant clonal expansion (22–24). None of these findings establishes that CHIP should guide treatment selection.

CHIP should therefore be analyzed by driver gene, clone size, tumor type, treatment modality, and detection context. Prospective studies with standardized sequencing, serial immune and clonal measurements, and prespecified tumor-specific analyses are needed before any phenotype can be used as an efficacy biomarker or toxicity-management tool. Until then, the most defensible clinical action is to recognize and confirm incidental CH variants when misattribution could change oncology care.
